# Supplementary material for: Evidence supported by Mendelian randomization: impact on inflammatory factors in knee osteoarthritis
Source: Front Med (Lausanne). 2024 May 28;11:1382836. doi: 10.3389/fmed.2024.1382836 (PMC11165061; doi:10.3389/fmed.2024.1382836)
Supplement: Supplementary file 1 [file Data_Sheet_1.ZIP › data/GCST90274836_5e6/836.forest.pdf]

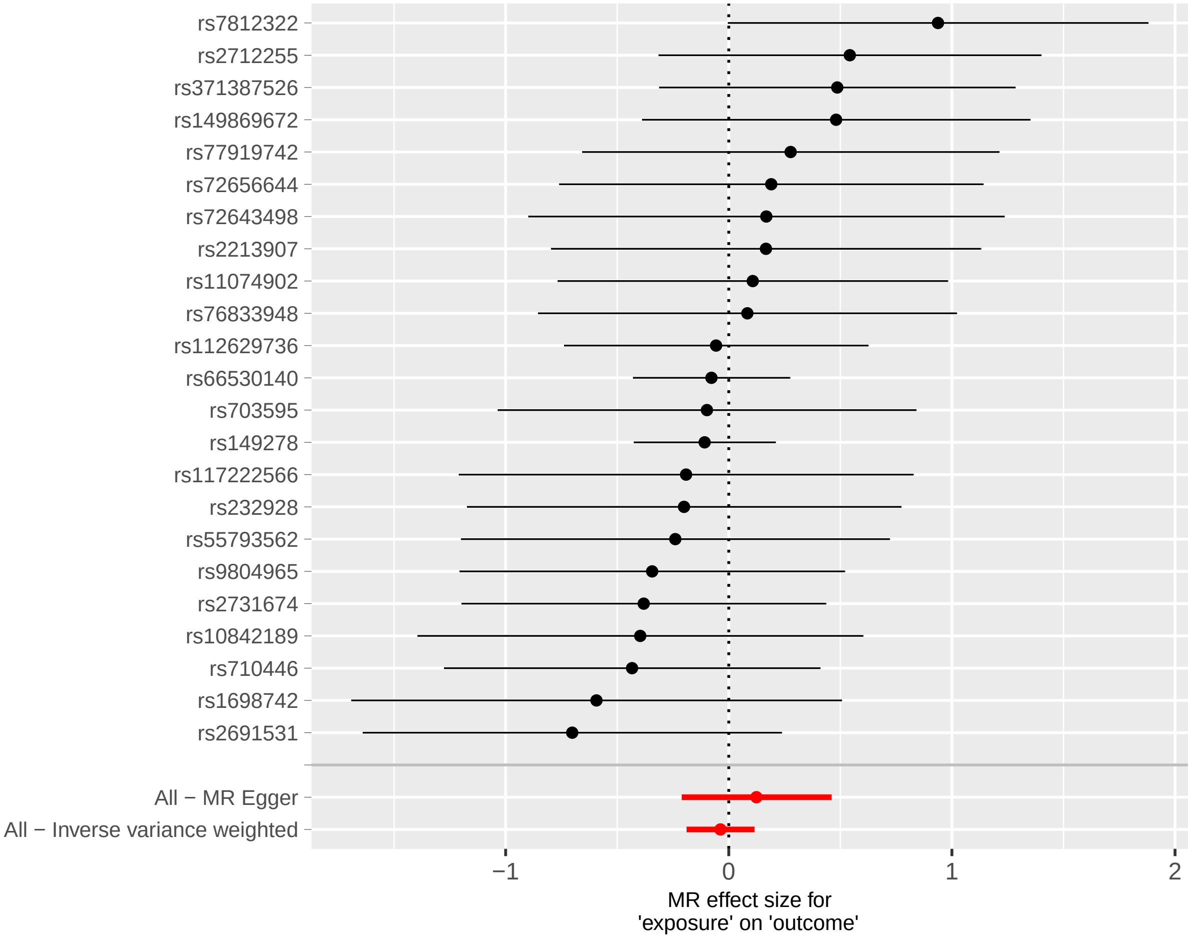
rs7812322
rs2712255
rs371387526
rs149869672
rs77919742
rs72656644
rs72643498
rs2213907
rs11074902
rs76833948
rs112629736
rs66530140
rs703595
rs149278
rs117222566
rs232928
rs55793562
rs9804965
rs2731674
rs10842189
rs710446
rs1698742
rs2691531
All − MR Egger
All − Inverse variance weighted
−1
0
1
2
MR effect size for
'exposure' on 'outcome'
